# CARD9 Signaling in Intestinal Immune Homeostasis and Oncogenesis

**DOI:** 10.3389/fimmu.2019.00419

**Published:** 2019-03-11

**Authors:** Lara Hartjes, Jürgen Ruland

**Affiliations:** ^1^Institute of Clinical Chemistry and Pathobiochemistry, School of Medicine, Technical University of Munich, Munich, Germany; ^2^German Cancer Consortium (DKTK), Heidelberg, Germany; ^3^German Center for Infection Research (DZIF), Munich, Germany

**Keywords:** CARD9, intestinal immune homeostasis, inflammatory bowel disease, intestinal inflammation, intestinal carcinogenesis, gut microbiome

## Abstract

Intestinal homeostasis requires a balanced interaction between the host innate immune system and the gut microbiota. A dysregulation of this interdependency can result in inflammatory bowel diseases (IBDs), and this dysregulation is a key pathogenic factor during the development of colorectal cancer. CARD9 is a central signaling molecule in the innate immune system, which is essential for host defense against infection. Moreover, polymorphisms in *CARD9* are key risk factors for IBD development, indicating that CARD9 signaling is critical for intestinal immune homeostasis. This review summarizes recent insights into the regulation of CARD9 signaling, its pathophysiological role during IBD development via effects on the microbiota and epithelial regeneration and the pro- and antitumor immune functions of CARD9 during intestinal carcinogenesis.

## Introduction

Homeostasis in the gastrointestinal tract depends on balanced interactions between the host immune system and the gut microbiome. The microbiome encompasses bacteria, fungi, and other microorganisms, and its composition is dynamically shaped by the host immune system, which continuously senses microbes and their metabolites and reacts with tolerogenic or stimulatory signals (1). Dysregulation of the immune-microbial balance due to the host's genetic composition, microbial dysbiosis, or environmental factors can cause inflammatory bowel diseases (IBDs), such as ulcerative colitis and Crohn's disease (2). Moreover, both overt chronic inflammation and smoldering inflammation are key risk factors for the development of gastrointestinal cancers (3). Therefore, understanding the reciprocal interaction between the host immune system and the intestinal microbiome is of great biomedical importance.

To sense microbial structures in the gut, innate immune cells are located in the lamina propria underneath the intestinal epithelium and in organized lymphoid structures. These cells are equipped with germline-encoded pattern recognition receptors (PRRs) that survey the intestinal environment and engage intracellular signaling pathways to induce the production of effector molecules that orchestrate local immunity. Importantly, a majority of genetic susceptibility loci that modulate the risk of developing IBD lie within genes involved in immune responses (4), which underscores the importance of immune signaling in IBD development. One very prominent IBD susceptibility gene whose key function in intestinal pathophysiology is being increasingly understood is *CARD9*, which encodes the innate immune signaling adapter protein CARD9 (4–8). This review will summarize recent insights into the molecular functions of CARD9 signaling and its role in intestinal inflammation and oncogenesis.

## Molecular Mechanisms of CARD9 Signaling

**Figure 1:**
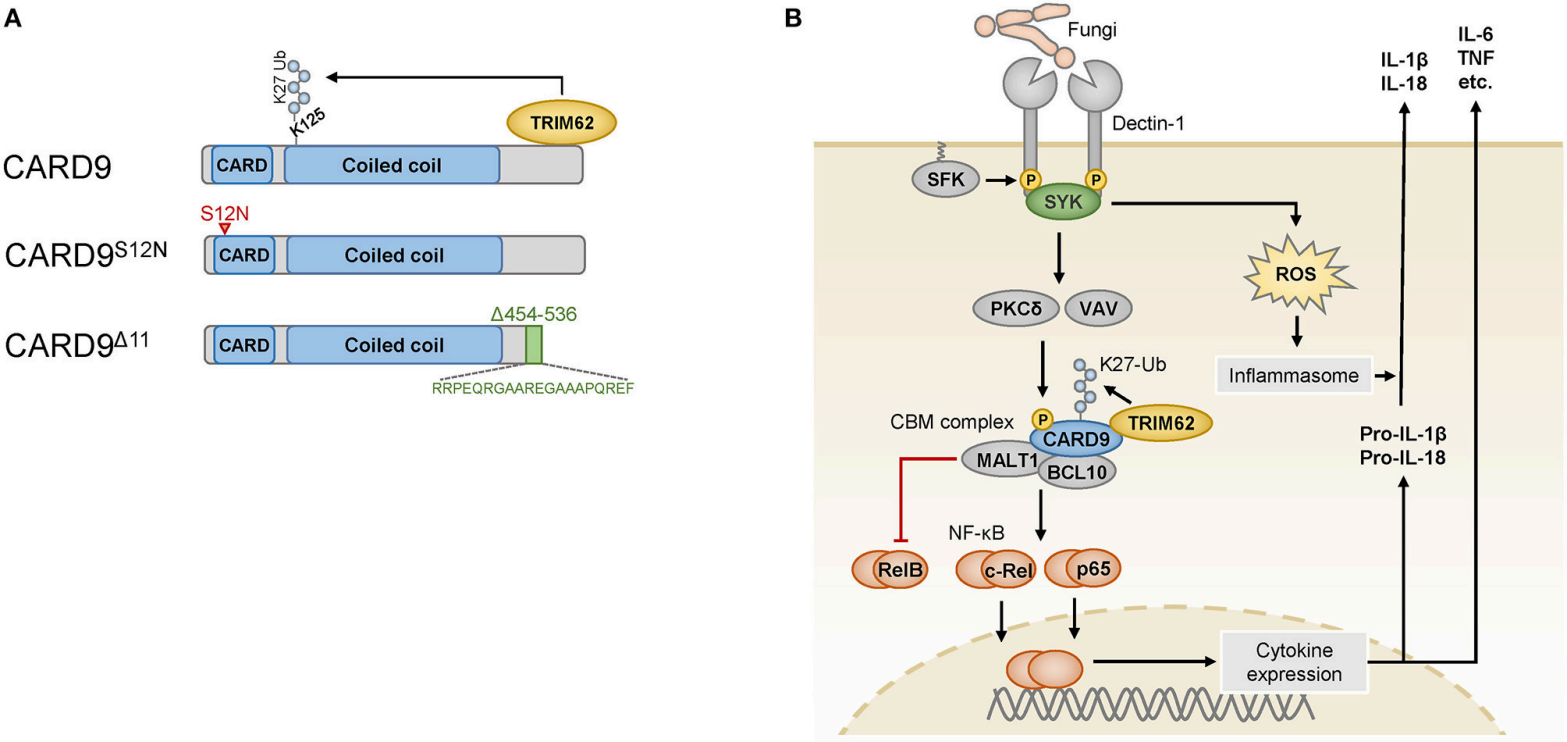
**(A)** The wild-type CARD9 protein consists of an N-terminal caspase activation and recruitment domain (CARD) followed by a coiled-coil domain and a C-terminal tail. The C-terminus mediates binding to the ubiquitin ligase TRIM62, which ubiquitinates CARD9 at K125. The CARD9^S12N^ variant encoded by an IBD risk-associated SNP carries an amino acid substitution in the N-terminal portion of the CARD domain (indicated by the red arrowhead). The rare variant CARD9^Δ11^ results from the skipping of exon 11, and instead of the amino acids 454–536, it bears a shortened and altered C-terminus (indicated in green) that is unable to bind to TRIM62. **(B)** Fungi are sensed by myeloid cells through Dectin-1, which is phosphorylated by SRC-family kinases (SFK) in its cytoplasmic portion upon ligand binding. The spleen tyrosine kinase (SYK) can recognize these phosphosites and activate the protein kinase C (PKC) δ and VAV proteins, which cooperate to phosphorylate CARD9, enabling it to assemble the CARD9-BCL10-MALT1 (CBM) complex. K27 ubiquitination of CARD9 by TRIM62 enables the activation of NF-κB, which consists of the transcriptionally active subunits c-Rel, p65 and RelB. RelB is counterregulated by the proteolytic activity of MALT1. Cytokines produced after stimulation of this pathway include IL-6, TNF and pro-IL-1β, which is further processed and released by inflammasomes that are triggered in a SYK- and reactive oxygen species (ROS)-dependent manner. Pro-IL-18 is similarly processed and released by the inflammasome.

CARD9 is selectively expressed in myeloid immune cells, including dendritic cells, macrophages, and neutrophils, and it is composed of an N-terminal caspase-activating and recruitment domain (CARD), a coiled-coil domain and a C-terminal tail with no specific domain structure (9) (Figure 1A). CARD9 can be activated by several plasma membrane-associated and intracellular PRRs (10, 11), and it is critical for the function of SYK-coupled C-type lectin receptors (CLRs). These CLRs, including Dectin-1 (12), Dectin-2 (13), and Mincle (14, 15), are key PRRs for the detection of commensal and pathogenic fungi but can also sense certain bacteria or danger signals released from dying host cells (16). The CLR-mediated activation of SYK leads to the activation of PKCδ via intermediate steps (Figure 1B), and PKCδ then directly phosphorylates CARD9 at T231 in the coiled-coil domain (17) in cooperation with VAV proteins (18). These molecular events enable CARD9 activation and the assembly of a BCL10- and MALT1-containing signaling platform, termed the CARD9-BCL10-MALT1 (CBM) complex, which serves as a scaffold to place ubiquitin modifiers and protein kinases in proximity to activate downstream effector pathways (9).

One major effector pathway of the CBM complex is the canonical IκB kinase (IKK)-mediated NF-κB pathway, which controls the expression of a variety of immune-regulatory factors. The NF-κB family consists of several subunits, of which p65, c-Rel, and RelB are transcriptionally active and form homodimers and heterodimers with other NF-κB proteins to initiate gene expression (19). To mediate NF-κB signaling, CARD9 recruits the ubiquitin ligase TRIM62, which binds to the C-terminal tail of CARD9 and mediates K27-linked ubiquitination of CARD9 on K125 in the coiled-coil domain (20) (Figure 1A). This modification is dispensable for the formation of the CBM complex but critical for the downstream activation of NF-κB (20). The CBM complex activates NF-κB dimers containing the subunits p65 or c-Rel (18), while MALT1 also functions as a protease that can inactivate the subunit RelB (21) to control distinct NF-κB subprograms. RelB is a subunit that is primarily activated by the noncanonical NF-κB pathway, and it can bind to the *IL5* promoter to activate *IL5* transcription in myeloid cells (22). Factors regulated by the canonical NF-κB pathway include pro-inflammatory cytokines, including TNF, IL-6 and the pro-inflammatory cytokine precursor pro-IL-1β, which is processed by inflammasomes into mature IL-1β. Inflammasomes are activated in response to fungal recognition in a SYK- and reactive oxygen species-dependent manner (23), and they can process additional cytokines of the IL-1 family, e.g., IL-18.

CARD9 signaling can also be activated by intracellular PRRs, such as the nucleotide-binding oligomerization domain-containing protein 2 (NOD2) (11), which is encoded by another important IBD risk gene, *NOD2* (4). In this pathway, NF-κB is activated independently of CARD9 by the interaction of NOD2 with RIPK2, whereas the interaction of NOD2 with CARD9 promotes the activation of the MAP kinases p38 and JNK, supporting pro-inflammatory cytokine production in response to stimulation with the bacterial cell wall component peptidoglycan or *Listeria monocytogenes* infection (11). Moreover, CARD9 is activated in response to the ligation of the nucleic acid receptors RIG-I (24) and RAD50 (25) after recognition of bacterial or viral nucleic acids, which also trigger the activation of the NF-κB pathway and the subsequent production of pro-inflammatory cytokines.

These CARD9-mediated signaling pathways are critical for host defense, particularly for protection against fungi, as CARD9-deficient mice and humans with *CARD9* loss-of-function mutations exhibit severe susceptibility to fungal infections caused by species such as *Candida albicans* (26) or dermatophytes (27), with invasive fungal growth into tissues (28). These protective functions are in part mediated by the critical role of CARD9 in inducing adaptive T_H_17 cell responses (26, 29), which exert defense mechanisms against fungi and other extracellular pathogens. Moreover, during fungal infection, CARD9 controls the induction of neutrophilic myeloid-derived suppressor cells (MDSCs) (30), which prevent damaging hyperinflammation by counterbalancing T and NK cell responses.

## *CARD9* Genetic Variants in Intestinal Inflammation

While a complete loss of CARD9 results in immunodeficiency, several single-nucleotide polymorphisms (SNPs) in the human *CARD9* gene are associated with inflammatory diseases, particularly with IBD (rs10781499 (4), rs10870077 (5), and rs4077515 (6, 7)). Non-coding or synonymous variants presumably influence the level of CARD9 expression (31, 32) and thereby modulate the strength of CARD9 signaling. In addition, the IBD-associated SNP rs4077515 encodes a missense CARD9 variant that carries an asparagine instead of a serine residue in the CARD domain in position 12 (CARD9^S12N^) (Figure 1A). Experiments with knock-in mice that carry the CARD9^S12N^-encoding allele have demonstrated that CARD9^S12N^ leads to aberrant activation of RelB and subsequent excessive production of IL-5 by myeloid cells in response to *A. fumigatus*, which skews T cell responses toward T_H_2 differentiation (22). These findings indicate that CARD9^S12N^ deviates physiological CARD9 signaling and suggest that the respective SNP in human IBD could potentially contribute to intestinal inflammation through related mechanisms, although this possibility remains to be investigated.

While the above-mentioned *CARD9* risk SNPs are relatively frequent and only moderately increase IBD risk (odds ratio (OR) ≈ 1.2), a rare splice site variant of CARD9 confers strong protection from IBD (OR ≈ 0.3–0.4) (8, 33). This specific variant, IVS11+1C>G, results in the skipping of exon 11, which leads to a CARD9 protein with a shortened C-terminal tail (CARD9^Δ11^) (Figure 1A). CARD9^Δ11^ is unable to bind TRIM62 and consequently fails to induce pro-inflammatory cytokines in myeloid cells after stimulation with fungal particles (20), which is thought to be responsible for the protective activity of CARD9^Δ11^ during IBD pathogenesis.

## CARD9 Signaling in Murine Models of Intestinal Inflammation

Based on the association of *CARD9* SNPs with IBD susceptibility, several research groups have started to investigate the role of CARD9 in murine models of experimental colitis (34, 35). In these studies, colitis was triggered by the administration of the chemical compound dextran sodium sulfate (DSS) via the drinking water (34, 35) or by oral inoculation with *Citrobacter rodentium* (34, 36). Both regimens damage the colonic epithelium and enable the entry of intestinal contents including bacteria and fungi into submucosal layers, triggering intestinal inflammation that leads to weight loss in the animals, followed by a recovery phase after DSS discontinuance in the DSS model (37, 38) or upon bacterial clearance in the *C. rodentium* model (34, 36).

**Figure 2:**
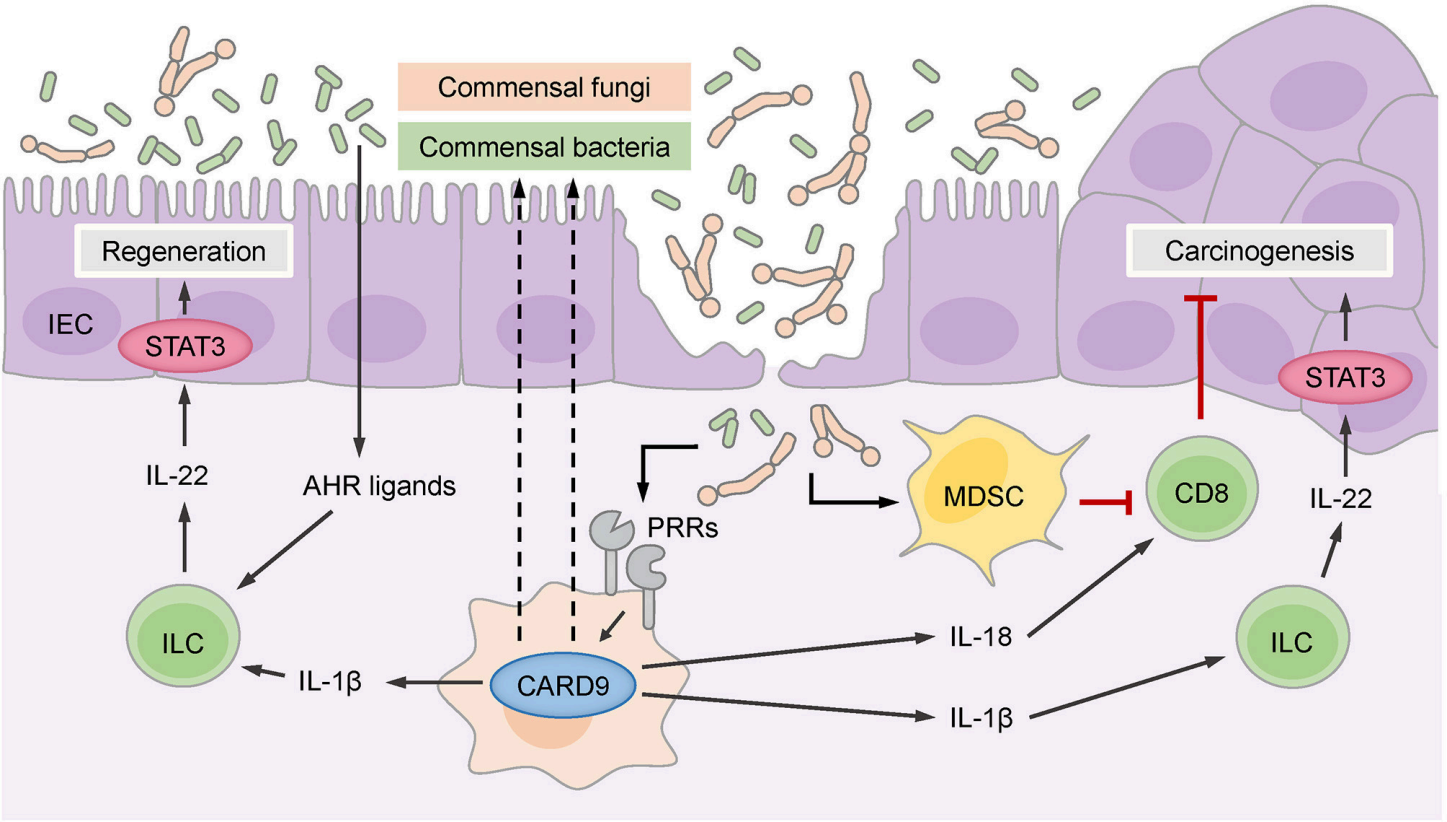
CARD9 controls the intestinal microbiome, which consists of commensal bacteria and fungi. Commensal gut bacteria supported by CARD9 provide aryl hydrocarbon receptor (AHR) ligands for the stimulation of innate lymphoid cells (ILCs) that secrete IL-22 to promote the regeneration of intestinal epithelial cells (IECs) through signal transducer and activator of transcription 3 (STAT3). ILCs are also stimulated by IL-1β, which is produced in a CARD9-dependent manner. CARD9 activated by pattern recognition receptors (PRRs) regulates commensal gut fungi that enter the damaged epithelium and promotes the accumulation of myeloid-derived suppressor cells (MDSCs). MDSCs inhibit CD8^+^ cytotoxic T cells that keep malignant IECs in check and thereby prevent carcinogenesis. CD8^+^ T cells are also stimulated by IL-18, which is secreted in response to commensal fungi in a CARD9-dependent manner. The CARD9-dependent IL-1β-IL-22 axis can support carcinogenesis through the stimulation of STAT3 in malignant IECs.

CARD9-deficient mice exhibited a defect in epithelial regeneration and a significant delay in weight gain during the recovery phase after acute colitis (34, 35). The deficiency of CARD9 affects primarily myeloid cells and cytokines produced by these cells, which secondarily act on other cell types in the intestinal environment. Based on this interplay, CARD9 deficiency results in reduced production of pro-inflammatory cytokines (IL-6, TNFα, and IFNγ) and T_H_17- and innate lymphoid cell (ILC)-related cytokines (IL-17A, IL-17F, and importantly IL-22) by leukocytes in the lamina propria and mesenteric lymph nodes (34, 35). This phenotype is in part caused by defective production of the chemokine CCL20, which is required to recruit T_H_17 cells and ILCs to sites of epithelial damage (34), and by the failure of CARD9-deficient myeloid cells to secrete optimal amounts of IL-1β, which is necessary to stimulate IL-22 production by ILCs (Figure 2) (35). IL-22 is a key cytokine that promotes recovery from DSS-induced or *C. rodentium*-induced inflammation. IL-22 binds to its receptor on intestinal epithelial cells (IECs), which drives their regenerative proliferation (39) and stimulates the expression of antimicrobial peptides for innate defense against intruding microbes. As a consequence of impaired IL-22 and IL-17 signals, the IECs in CARD9-deficient mice do not receive the proper pro-proliferative signals for recovery (35), and they fail to produce sufficient amounts of antimicrobial peptides (34). Apart from controlling gut microbes through the epithelial compartment, CARD9-expressing myeloid cells also provide signals that support humoral responses to *C. rodentium* (36), although the exact mechanisms of this interaction remain to be investigated.

Additional work demonstrated that CARD9 also shapes the composition of the gut microbiome and affects both fungal and bacterial communities (40). Under steady-state conditions, the fungal burden is increased in CARD9-deficient mice, and during colitis, the fungal microbiome changes more substantially in CARD9-deficient mice than in wild-type mice. Similarly, at baseline, the composition of the bacterial microbiome is disturbed in CARD9-deficient mice and exhibits decreased stability during DSS colitis. Underrepresented bacteria in CARD9-deficient mice include, for example, species from the *Adlercreutzia* genus and *Lactobacillus reuteri*. Under steady-state conditions, this dysbiotic microbiota of CARD9-deficient mice exhibits a striking failure to metabolize tryptophan into ligands of the aryl hydrocarbon receptor (AHR) transcription factor (Figure 2), which is crucial for the expression of IL-22 by T_H_17 cells (39). This failure to produce AHR ligands has also been linked to impaired IL-22 production and delayed epithelial restitution in CARD9-deficient mice (40). Together, these experimental studies revealed that CARD9 signaling within the myeloid compartment affects epithelial regeneration during the recovery from colitis by triggering ILC and T_H_17 responses, in part by directing the microbiota toward AHR ligand-producing species. These pathways are presumably also important for the pathogenesis of IBD in patients with CARD9 risk variants, as a deficiency in AHR ligands is observed in the microbiota of IBD patients with the *CARD9* risk SNP rs10781499 (40).

## CARD9 in Immune Environments in Colorectal Cancer

The inflammatory pathways that stimulate the regenerative expansion of normal IECs are also frequent drivers of malignant proliferation and survival in transformed epithelial cells during colorectal cancer pathogenesis (3). However, in addition to providing pro-inflammatory, tumor-promoting effects, immune cells also play a protective role in colorectal cancer, which is largely executed via the antitumor activity of cytotoxic CD8^+^ T cells, whose presence within the tumor tissue is one of the strongest prognostic markers for a positive clinical outcome in colorectal cancer patients (41). Recent studies revealed that CARD9 signaling can contribute to both tumor-promoting and tumor-suppressive immunity in the intestine, and these distinct effects are strongly influenced by the composition of the microbiota (35, 42, 43).

To explore the role of CARD9 in colitis-associated cancer (CAC) independent groups used CARD9-deficient mice in the AOM/DSS model (35, 42–44). In this model, the administration of the carcinogen azoxymethane (AOM) initiates mutations in IECs, and subsequent cycles of DSS administration trigger inflammation that promotes epithelial dysplasia through a variety of immune cell-derived mediators, growth factors and cytokines. In wild-type mice, this treatment protocol provokes the formation of multiple polyploid lesions in the colon and rectum, with histological scores from low to high grade, but invasive dysplasia rarely occurs in this context.

To minimize the effects of the microbiota on tumor development, Bergmann et al. co-housed CARD9-deficient mice and littermate controls from birth on and during the AOM/DSS treatment protocol (35). Under these conditions, CARD9-deficient mice displayed smaller polyps in the distal colon than wild-type mice, although the total number of polyps did not differ between CARD9-deficient and wild-type mice. Moreover, the rate of tumor cell proliferation was decreased in CARD9-deficient mice, and the tumor cell-intrinsic activation of STAT3 was substantially reduced. STAT3 is a key transcription factor that drives the proliferation and survival of malignant cells during CAC pathogenesis (45) and is activated by several cytokines and growth factors, including IL-22 (39). These experiments indicate that CARD9 signaling in the immune environment of developing colorectal cancers can trigger the tumor-promoting STAT3 pathway within tumor cells, presumably via ILC-mediated IL-22 production, comparable to its role during epithelial regeneration after acute colitis (34, 35).

Two subsequent studies single-housed CARD9-deficient and wild-type animals to dissect the microbiota-dependent influences on CAC development using the AOM/DSS model (42, 43). Under these conditions, CARD9-deficient mice develop more and larger tumors than wild-type mice, and these effects were attributed to an aberrant fungal microbiome and secondary effects of fungi on antitumor T cells (42, 43). Wang et al. found that several fungal species, particularly *Candida tropicalis*, were enriched in the intestinal flora of CARD9-deficient mice (43) and interestingly also in the feces of colorectal cancer patients (43). Moreover, monocolonizing germ-free mice with *C. tropicalis*, but not with the irrelevant fungal species *Saccharomycopsis fibuligera*, increased their susceptibility to CAC in the AOM/DSS model, indicating that this species can support cancer development (43). Mechanistically, Wang et al. proposed that specific fungi promote the accumulation and activation of granulocytic MDSCs in the colonic lamina propria, which suppress the proliferation and effector functions of T cells and thereby negatively interfere with T cell-mediated antitumor immunity and enable enhanced colorectal cancer growth (Figure 2). Since the fungal load in the feces of colorectal cancer patients positively correlates with MDSC frequencies in the tumor, this pathway could also play an important role in clinical settings (43).

Similarly, Malik et al. observed an increased tumor burden in CARD9-deficient mice using the AOM/DSS model under single-housing conditions (42), but they propose an additional mechanism. While this group also demonstrates that CARD9 signaling shapes the composition of fungal communities and T cell immunity in the colon, they show that CARD9 controls the IL-1 family cytokine IL-18 through a canonical SYK-dependent pathway in myeloid cells in the early phase of the AOM/DSS treatment protocol (42) (Figure 2). IL-18 can maintain intestinal epithelial integrity during inflammation and stimulate IFNγ and FASL expression by T cells (42, 46), supporting antitumor immunity during CAC development. Consistent with this model, supplementation with exogenous IL-18 restored T cell function in CARD9-deficient mice during CAC, ameliorated the exacerbated intestinal inflammation and reduced tumor development (42).

Together, these two studies reveal previously unknown functions of CARD9 in the control of fungal communities that initiate MDSCs and stimulate IL-18 secretion, both of which regulate the function of antitumor CD8^+^ T cells during CAC development (Figure 2). Nevertheless, it remains unclear to what extent the overall fungal load determines CAC outcome, as the pharmacological depletion of fungi can on one hand reduce MDSC frequencies and thus enhance antitumor T cell responses in CARD9-deficient mice (43) but on the other hand result in a lack of IL-18 signals for stimulation of antitumor T cells (42).

## Conclusion and Perspective

The human *CARD9* locus has been a known IBD susceptibility factor for more than 10 years, and during that time, the basic mechanisms of CARD9 signaling have been elucidated (9). In IBD, both risk-promoting and protective CARD9 variants have been identified, and based on an understanding of the protective CARD9 variant, which is defective in the CARD9-TRIM62 interaction, small-molecule inhibitors that block the CARD9-TRIM62 interaction have been developed that might be useful for IBD treatment (47). Additional pathomechanisms of CARD9 signaling during IBD are alterations in regenerative IL-22 signaling via the effects of CARD9-expressing myeloid cells on ILCs (34, 35) or via the effects of CARD9 on the control of the microbiota (40, 42, 43), which could also provide new strategies for IBD treatment. The key role of CARD9 in intestinal immune homeostasis is also underscored by the influence of CARD9 signaling on colorectal cancer development. Here, depending on the composition of the microbiota, particularly the fungal communities, CARD9 can play a pro-tumorigenic role by triggering inflammation-induced oncogenic STAT3 activation within malignant cells or a tumor-suppressive function by promoting antitumor T cell responses through various mechanisms. How the balance between these CARD9-mediated pro- and antitumor immune functions is precisely tuned requires further investigation. Such studies are expected to provide further insights into the complex interplay among the immune system, the microbiota, tissue homeostasis and cancer development, as well as potential rational targets for therapeutic interventions.

## Author Contributions

All authors listed have made a substantial, direct and intellectual contribution to the work, and approved it for publication.

### Conflict of Interest Statement

The authors declare that the research was conducted in the absence of any commercial or financial relationships that could be construed as a potential conflict of interest.
